# Validation of the FACT-G7 in patients with hematologic malignancies

**DOI:** 10.3389/fonc.2023.1183632

**Published:** 2023-08-10

**Authors:** Xinwen Du, Ling Mao, Yamei Leng, Fengjiao Chen

**Affiliations:** Department of Hematology, West China Hospital/West China School of Nursing, Sichuan University, Chengdu, China

**Keywords:** FACT-G7, hematologic malignancy, measurement, quality of life, reliability, validity

## Abstract

**Background:**

It is essential to evaluate the quality of life in patients with hematologic malignancies to reflect the therapeutic effect and prognosis, but lengthy assessments are often burdensome. The 7-Item Functional Assessment of Cancer Therapy-General (FACT-G7) is a brief, easy, and rapid index for evaluating quality of life. Nevertheless, there is no report about its application in Chinese patients with hematologic malignancies.

**Objective:**

The purpose of this study was to validate the Chinese version of the FACT-G7 for patients with hematologic malignancies.

**Methods:**

This study is a cross-sectional study. A total of 855 patients with hematologic malignancies completed the Functional Assessment of Cancer Therapy-General (FACT-G) and were scored the Eastern Cooperative Oncology Group Performance Status (ECOG-PS) by nurses. Cronbach’s alpha, confirmatory factor analyses, Pearson’s correlation, and one-way analysis of variance were conducted to evaluate internal consistent reliability, structural validity and concurrent validity.

**Results:**

The FACT-G7 showed acceptable internal consistency, as indicated by a Cronbach’s alpha of 0.73. The confirmatory factor analyses test for single-factor model fit for the FACT-G7 scale was almost adequate. The satisfactory correlations between the FACT-G7 and the FACT-G and its subscales, and ECOG-PS groups differed in FACT-G7 scores demonstrating concurrent validity.

**Conclusion:**

This study suggested that the Chinese version of the FACT-G7 provides a useful and rapid measure for assessing quality of life in Chinese patients with hematologic malignancies, which providing a reference for further evaluation and care.

## 1 Introduction

Hematologic malignancies are characterized by highly malignant and differentiation disorders, among which Hodgkin’s lymphoma, non-Hodgkin’s lymphoma, acute leukemia, and myeloma are the most common, accounting for 73.5% of the burden (1). Leukemia comprises 3.1% of all cancer deaths worldwide, and approximately 0.54 million new cases of non-Hodgkin’s lymphoma were diagnosed worldwide in 2020 (2). In China, the incidence of leukemia and lymphoma was 6.21/10^5^ and 6.50/10^5^, and the mortality was 4.04/10^5^ and 3.73/10^5^ respectively (3). Hematologic malignancies pose a serious threat to human health around the world.

As hematologic malignancy treatments have extended overall and progression-free survival over the past two decades, both patients and medical personnel have turned their attention toward improving quality of life (4, 5). Patients with hematologic malignancies often experience significant physical symptoms such as nausea, vomiting, pain, fatigue, bleeding, infection, and neuropathy related to progressive cancer and anticancer treatments, and patients frequently experience psychological distress due to long-term disease burden, both of which have negative impacts on the quality of life of hematologic malignancy survivors (6, 7).

Quality of life has emerged as an increasingly important critical target of efficacy assessment in hematologic malignancies to be considered alongside survival (8, 9). Although there are numerous instruments available to measure quality of life in patients with cancer, the gold standard among quality of life assessments is lacking (10). Patients with hematologic malignancies often experience multiple symptoms, especially fatigue (11). Compared with patients with solid tumors, those with hematologic malignancies experience higher odds of fatigue (12), with 30%-80% of patients reporting fatigue during the disease (13, 14). Thus, the length of the questionnaire may be a more significant concern or burden for them. Given the physical and emotional conditions of hematologic malignancy patients, quality of life assessments need to be brief, rapid, facile, and still able to capture the most relevant patient issues.

The 7-Item Functional Assessment of Cancer Therapy-General (FACT-G7) is a brief index comprising 7 high-priority Functional Assessment of Cancer Therapy-General (FACT-G) scale items for evaluating quality of life in advanced cancer patients (8). The original FACT-G7 scale showed good internal consistency and criterion validity (8). Studies (15, 16) have demonstrated that the FACT-G7 exhibited good test–retest reliability, fit for a single-factor structure, convergent, and discriminant validity, and responsiveness to change in the quality of life over time after interventions. This rapid questionnaire takes only a few minutes to complete and requires little assistance (8). Even with multiple symptoms, including fatigue, the FACT-G7 scale may be feasibly applied by hematologic malignancy patients themselves. Therefore, the scale is potentially useful in the assessment of quality of life and the evaluation of therapeutic effectiveness among hematologic malignancy patients. However, there is currently a lack of reports on the application of the FACT-G7 scale in a sample of Chinese patients with hematologic malignancies.

Thus, this study aimed to validate the original FACT-G7 scale for Chinese patients with hematologic malignancies.

## 2 Materials and methods

### 2.1 Study design and setting

This cross-sectional, descriptive study was conducted in West China Hospital, a 4300-bed tertiary teaching hospital affiliated with Sichuan University and the leading medical center in southwestern China, from June 2019 to November 2022. This study is reported following the Strengthening the Reporting of Observational studies in Epidemiology (STROBE) guideline (17) as shown in **Supplementary Material 1**.

### 2.2 Participants

Hospitalized patients in the hematology ward were recruited. Patients were included if they were (1) at least 18 years old; (2) diagnosed with hematologic malignancy; (3) receiving anticancer therapy; (4) had adequate reading and writing ability to finish the survey; and (5) willing to participate in the study. Of the 1216 patients we approached, 1045 met the eligibility criteria and 868 gave their informed consent. Ultimately, a total of 868 questionnaires were collected and 13 were excluded as invalid because the answers to all items were consistent.

### 2.3 Measures

The FACT-G is a widely used instrument in oncology quality of life assessment and consists of 27 items in 4 subscales: physical well-being, social/family well-being, emotional well-being, and functional well-being. Each item was scored from 0 (not at all) to 4 (very much) (18). Total scores range between 0 and 108, with higher scores reflecting better quality of life (18). The Chinese version of the FACT-G has been validated (19). We obtained the Chinese version of the FACT-G from the official website (https://www.facit.org) and acquired permission to use it.

The FACT-G7 is a rapid, brief quality of life index consisting of 7 high-priority FACT-G items from the physical well-being subscale (fatigue, pain, and nausea), emotional well-being subscale (worry about condition worsening), and functional well-being subscale (enjoyment of life, contentment with quality of life, and sleep) (8). Total scores range between 0 and 28, and higher scores indicate better quality of life (8). Previous studies have demonstrated that the FACT-G7 is a rapid index for evaluating quality of life in advanced cancer patients with good reliability and validity (8, 16). We also obtained the Chinese version of the FACT-G7 from the official website (https://www.facit.org) and acquired permission to use it.

The Eastern Cooperative Oncology Group Performance Status (ECOG-PS) is the most common scale to quantify performance status and is considered a simple tool to use in daily clinical practice (20). It ranges from 0 to 5, and a higher value reflects a lower performance status (21). ECOG PS of 0 indicates fully active; a value of 1 indicates restricted in strenuous activity but ambulatory; a value of 2 indicates ambulatory and capable of all self-care but unable to carry out any work activities, up and approximately > 50% of waking hours; a value of 3 indicates confined to bed or chair for more than 50% of the time with only limited self-care; a value of 4 indicates disabled and bedridden; and 5 represents death (21).

### 2.4 Procedure

Two research assistants were trained to conduct the investigation and data collection. All hospitalized patients in the hematology ward were approached and assessed for eligibility. Eligible patients were informed about the study, and patients who gave written informed consent were asked to complete a self-report questionnaire.

### 2.5 Statistical analysis

SPSS version 21.0 (Statistical Package for the Social Sciences; IBM Corp., Armonk, NY) and AMOS version 26.0 (IBM Corp., Armonk, NY, US) were used for statistical analysis. Missing data is acceptable as long as the items answered in the subscale exceed 50% and the overall scale response rate is more than 80% (https://www.facit.org). It can be prorated using the average of other answers in the subscale. Continuous variables were summarized using means with standard deviations (SDs). Categorical variables were summarized using frequencies with proportions. Details of the specific validity and reliability testing methods are as follows.

Internal consistency: Internal consistency was evaluated using Cronbach’s alpha coefficient (Cronbach’s α), and a Cronbach’s α of 0.7 and above represents acceptable consistency (22).

Structural validity: Confirmatory factor analysis (CFA) was conducted to test the structural validity. The estimation of model parameters and fit indices for the CFA was conducted according to the maximum likelihood method. The following indices for model fit were used: a chi-square/df ratio (χ2/df), root mean square error of approximation (RMSEA), the goodness of fit index (GFI), comparative fit index (CFI), and incremental fit indices (IFI). χ2/df ≦ 2.0 indicates a good fit, < 3.0 indicates a reasonable fit, GFI, CFI, and IFI > 0.90 indicate a good fit, and > 0.80 indicates a reasonable fit, while RMSEA < 0.05 indicates a good fit, and < 0.08 indicates a reasonable fit (23, 24).

Concurrent validity: Pearson correlations of the FACT-G and its subscales with the FACT-G7 were performed to assess concurrent validity. The correlation values for convergent validity were categorized as follows: small correlation (r < 0.40), moderate correlation (r = 0.40-0.70), and strong correlation (r > 0.7) (22). Furthermore, we conducted a one-way analysis of variance to evaluate whether ECOG PS groups significantly differed in FACT-G7 scores. Consistent with prior research (8, 16), we hypothesized that the scores of the FACT-G and its subscales (except for the FACT-G social/family subscale) were at least moderately correlated with the FACT-G7. We also hypothesized that patients with lower ECOG PS ratings would report higher FACT-G7 scores.

## 3 Results

### 3.1 Missing values

Of the 855 FACT-G questionnaires, 703 (82.2%) had missing data, the vast majority (680, 96.7%) appeared in the optional entry “I am satisfied with my sex life”, and the remaining items with more missing values were “I am able to work (include work at home)”, with 44 (5.1%) missing, and “I feel close to my partner (or the person who is my main support)”, with 12 (1.4%) missing. For the FACT-G7 questionnaire entries, only 14 (1.6%) had missing data, and the “I am sleeping well” entry was the item with the most missing values, with 4 (0.5%) missing. According to the scoring guidelines of the questionaries (https://www.facit.org), since the items answered in the subscale exceed 50% and the overall scale response rate is more than 80%, the missing values in this study were considered acceptable.

### 3.2 Participant characteristics

The sociodemographic and clinical characteristics of the patients are presented in **Table 1**. The participates’ mean age was 48 years (standard deviation = 16.51), and their ages ranged from 18 to 91 years. Most of the participants were male (50.8%), married (80.8%), and had received middle school or lower levels of education (47.8%). The participants were diagnosed with various types of hematologic cancer, most of which were acute myeloid leukemia (42.0%) and multiple myeloma (24.4%).

**Table 1:** Demographic and clinical characteristics of participants (N = 855).

Variable	Mean ± SD or n (%)
**Age (years)**	48.00 ± 16.51 (range, 18-91)
Sex	
Female	421 (49.2%)
Male	434 (50.8%)
Education	
Primary school or below	168 (19.6%)
Middle school	241 (28.2%)
High school	154 (18.0%)
Associate degree	142 (16.6%)
Bachelor’s degree or above	150 (17.5%)
Marital status	
Married	691 (80.8%)
Single/divorced	164 (19.2%)
Diagnosis	
Acute myeloid leukemia	359 (42.0%)
Acute lymphoblastic leukemia	143 (16.7%)
Multiple myeloma	209 (24.4%)
Hodgkin lymphoma	10 (1.2%)
Indolent non-Hodgkin lymphoma	26 (3.0%)
Aggressive non-Hodgkin lymphoma	108 (12.6%)
Time since diagnosis (months)	
≤1	384 (44.9%)
1~6	212 (24.8%)
6~12	90 (10.5%)
>12	169 (19.8%)
Treatment status	
Anti-cancer drug treatment	832 (97.3%)
Radiotherapy	2 (0.23%)
HSCT	20 (2.34%)
CAR-T therapy	1 (0.12%)
Relapsed or refractory	
Yes	101 (11.8%)
No	754 (88.2%)
FACT-G	74.33 ± 16.05
FACT-G7	18.78 ± 5.09
ECOG PS	
0	254 (29.7%)
1	547 (64.0%)
2	43 (5.0%)
3	11 (1.3%)

SD, Standard deviation; n, Portion of total sample; %, Percentage of sample; HSCT, Hematopoietic stem cell transplantation; CAR-T, Chimeric antigen receptor T cell; ECOG PS, Eastern Cooperative Oncology Group Performance Status; FACT-G, Functional Assessment of Cancer Therapy-General.

### 3.3 Internal consistency

**Table 2** shows the internal consistency and item-total correlation of the FACT-G7 scale. The internal consistency analysis showed a Cronbach’s alpha of 0.73 for the FACT-G7. All the items were correlated to the scale’s total score (range, 0.34-0.56). In all cases, deleting an item would result in a slight reduction in the corresponding Cronbach’s α (range, 0.68–0.72).

**Table 2:** Internal consistency and item-total correlation of FACT-G7 items (N = 855).

Item	Item-total correlation	Cronbach’s alpha if item is deleted	Cronbach’s alpha
**I have a lack of energy**	0.43	0.70	0.73
**I have nausea**	0.38	0.71	
**I have pain**	0.40	0.71	
**I worry that my condition will get worse**	0.34	0.72	
**I am able to enjoy life**	0.49	0.68	
**I am sleeping well**	0.48	0.69	
**I am content with the quality of my life right now**	0.56	0.67	

### 3.4 Structural validity

**Figure 1:**
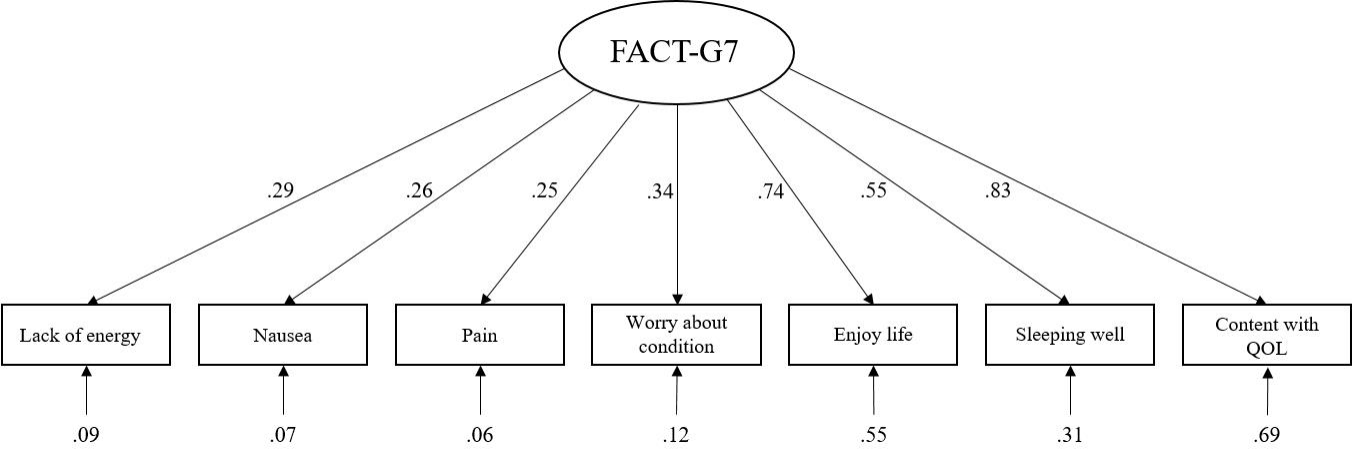
Standardized factor loadings for the single-factor model. QQL, Quality of life.

**Figure 1** indicates the estimated standardized factor loadings for the FACT-G7 scale model, and all factor loadings were significant (P < 0.001). The test for model fit for the FACT-G7 scale was almost adequate, with the following fit indices: χ2(11) = 71.87, p<0.001; RMSEA = 0.08, p = 0.002, 90% CI = 0.06-0.10; GFI = 0.98; CFI = 0.95; ILI = 0.95.

### 3.5 Concurrent validity

A statistically significant correlation was observed between the FACT-G7 score and the FACT-G total score (r = 0.87, P < 0.001). Large positive correlations with the FACT-G physical and functional subscales, moderate correlations with the emotional subscale, and small correlations with the social/family subscale were observed in **Table 3**, implying that concurrent validity was satisfied.

**Table 3:** Correlations between FACT-G7 Scores and FACT-G (N = 855).

Items	Correlations	P-value
**PWB**	0.76	< 0.001
**SWB**	0.23	< 0.001
**EWB**	0.64	< 0.001
**FWB**	0.77	< 0.001
**FACT-G total**	0.87	< 0.001

FACT-G, Functional Assessment of Cancer Therapy-General; PWB, Physical well-being; SWB, Social/family well-being; EWB, Emotional well-being; FWB, Functional well-being.

As shown in **Table 4**, ECOG PS rating groups significantly differed in FACT-G and FACT-G7 scores (P < 0.001), and scores on the FACT-G and FACT-G7 decreased with declining ECOG PS rating groups. Pairwise group comparisons indicated that the score differences between ECOG 0 rating, ECOG 1 rating and ECOG 2 rating for the FACT-G total score and FACT-G7 exceeded the respective meaningful difference thresholds (P < 0.05).

**Table 4:** Comparisons of ECOG PS groups in FACT-G7 and FACT-G scores (N = 855).

Scales	ECOG PS				F	P-value	Partial η² ^a^	Multiple Comparisons
	0	1	2	3				
**FACT-G7**	20.91 ± 4.44	18.16 ± 5.02	14.98 ± 5.11	15.27 ± 3.90	30.00	< 0.001	0.096	EOCG_0_ > EOCG_1_ > EOCG_2_
**FACT-G**	80.47 ± 15.24	72.33 ± 15.73	66.18 ± 14.83	63.86 ± 10.52	21.97	< 0.001	0.072	EOCG_0_ > EOCG_1_ > EOCG_2_

ECOG PS, Eastern Cooperative Oncology Group Performance Status; FACT-G, Functional Assessment of Cancer Therapy-General; EOCG_0/1/2_, Eastern Cooperative Oncology Group Performance Status = 0/1/2.

a Partial η²: Measure of effect size, recommended interpretation: 0.01=small, 0.06=medium, 0.14=large.

## 4 Discussion

This study demonstrated evidence to support the reliability and validity of the FACT-G7 for measuring quality of life among Chinese people suffering from hematologic malignancies. Adequate internal consistency, a good fit for a single-factor structure, significant correlations with FACT-G domains and summary composite scores, and differentiating ECOG levels of patients, demonstrated the availability of a valid and reliable tool that makes it less burdensome for hematologic malignancy patients and provides comprehensive general information about quality of life.

Compared with the FACT-G questionnaire, the FACT-G7 had a higher degree of completion, which is largely resulting from the optional entry in the FACT-G. Another item with more missing values was “I am able to work (include work at home)”, which may be related to the uncertainty of working ability caused by negative illness perception in cancer survivors (25, 26). In addition, Kang’s study has shown that cancer stigma may cause resistance to work in patients (27).

Reliability was supported by high internal consistency which was assessed by calculating Cronbach’s alpha. Our study showed that the Cronbach’s alpha of the FACT-G7 was 0.73, which is higher than the acceptable coefficient of 0.70, and the item-total correlation coefficients ranged from 0.34 (worry about condition worsening) to 0.56 (contentment with quality of life) (both p < 0.01). In addition, Cronbach’s alpha if an item was deleted (0.67-0.72) in the FACT-G7 indicated that each item greatly contributed to the total scale. These results are similar to those previously reported for the validation study in cancer patients (8, 16), and indicate a high internal consistency reliability of the FACT-G7.

CFA was performed to confirm structural validity, and the fit indices except chi-square reported in this study were satisfactory. The result showed that the chi-square was statistically significant, suggesting that there were some differences between the data and the single-factor model. In fact, the sample size is a great factor affecting the chi-square goodness-of-fit test (28). With large sample sizes, such as the sample size of 855 cases in this study, even a very small difference between the hypothesized model and the observed data may result in statistical significance. Therefore, the other most common fit indices, including RMSEA, GFI, CFI, and ILI reported in this study suggest a good model fit.

Satisfactory correlations between the FACT-G7 and the FACT-G and its subscales and ECOG PS were found in our study, indicating good concurrent validity. Consistent with those reported in previous studies (8, 16), the FACT-G7 scale was highly correlated to the FACT-G total score, strong correlations with the FACT-G physical and functional subscales, and weaker relations with the social/family subscale related to the fact that most FACT-G7 items come from the FACT-G physical health and functional health subscale. Furthermore, the FACT-G7 was able to differentiate the groups defined by ECOG PS score, with a medium effect size. The results showed that the sample did not include patients whose ECOG PS value was above 3, probably because patients with such poor performance status were unable to participate in this survey. Through multiple comparisons, we found that FACT-G7 could well differentiate patients with ECOG PS of 0-2, and there was no significant difference in FACT-G7 scores between the value of 2 and the value of 3, which suggested that it is not enough to use FACT-G7 to evaluate the quality of life of patients with poor quantify performance status (such as ECOG PS score above 2). This may be resulted from the low proportion of patients with ECOG PS of 3. On the other hand, the original intention of the FACT-G7 design is rapid monitoring of the symptom/concern burden and quality of life across a wide range of cancer patients and providing a reference for further assessment and care (8).

This study has several limitations. Although the sample size had adequate power for the validation study, the single-site investigation of only hospitalized patients, uneven distribution of disease diagnoses, younger age groups, and the vast majority of patients were with ECOG-PS 0 and 1 are sample limitations of this study. Another limitation of this study is the lack of a longitudinal study to explore how patients’ quality of life changes throughout treatment to evaluate the test-retest reliability and responsiveness of the FACT-G7. The subsequent longitudinal study that includes more representative sample is warranted to verify the study results.

In conclusion, the results of this study support the feasibility, reliability, and validity of the Chinese version of the FACT-G7 in the measurement of quality of life in patients with hematologic malignancies. The FACT-G7 provides a useful and rapid measure for assessing quality of life in patients with hematologic malignancies, which would assist clinicians and researchers in evaluating the quality of life of patients who are too distressed to tolerate of lengthy instruments (e.g., most patients with hematologic malignancies) in a short time.

## Data availability statement

The raw data supporting the conclusions of this article will be made available by the authors, without undue reservation.

## Ethics statement

This study was approved by the Biomedical Ethics Committee of the West China Hospital, Sichuan University (Approval number: 2019769). The patients/participants provided their written informed consent to participate in this study.

## Author contributions

Conceptualization: FC, LM, and XD. Methodology: FC, LM, and XD. Investigation: LM, YL, and XD. Data curation: LM. Formal analysis: XD. Writing—original draft preparation: XD. Writing—review and editing: FC. Supervision: YL. Funding acquisition: FC. All authors have read and agreed to the published version of the manuscript.
